# Effects of a Lottery Incentive on STI/HIV Incidence Among Female Sex Workers in Tanzania: Outcomes of Rewarding STI Prevention and Control in Tanzania (RESPECT-2)

**DOI:** 10.1007/s10461-025-04822-8

**Published:** 2025-09-01

**Authors:** Marianna Balampama, Damien de Walque, William H. Dow, Rebecca Hémono

**Affiliations:** 1 Independent Scholar, Dar-Es-Salaam, Tanzania; 2 Development Research Group, The World Bank, Washington, DC 20433 USA; 3 School of Public Health, University of California, Berkeley, Berkeley, CA USA

**Keywords:** Female sex worker, Lottery, Incentive, HIV

## Abstract

Female sex workers (FSW) are a key population who experience a disproportionately high burden of HIV and sexually transmitted infections (STIs). A growing body of evidence suggests that financial incentives can reduce risky sexual behavior and reduce HIV/STI incidence, however few studies have examined a lottery-based incentive mechanism or been conducted with FSW. We examined the effect of a lottery intervention on combined HIV/HSV2 incidence among FSW. The RESPECT II trial was an unmasked, two-arm, parallel group randomized controlled trial conducted in Dar es Salaam, Tanzania (AEA RCT registry: AEARCTR-0002677). Individuals who were ≥ 18 years of age, HIV-negative, not currently pregnant, exchanged sex for money in the past six months, and living in Dar es Salaam were eligible. Participants were randomized in a 1:1 ratio to the basic test group (control) which included testing and counseling for HIV and biweekly text messages with information on safe sex practices, or to the lottery group, which provided the basic test group intervention plus entry into a weekly lottery with a 100,000 TZS (~ $50 USD) reward offered to ten randomly selected participants, conditional on negative test results for syphilis and trichomonas. The primary outcome was combined HIV/HSV2 incidence after 36 months. Between August 2018-February 2019, 2,489 individuals screened for eligibility and 2,206 were enrolled in the trial and randomized. Participants were followed for up to 36 months; 1089 (49.4%) were lost to follow-up at endline and 1,117 were included in the primary intent to treat analysis (609 lottery, 508 control). At 36 months, there was no effect of the lottery intervention on the incidence of combined HIV/HSV2 (unadjusted RD: − 0.006, 95% CI − 0.05, 0.04; adjusted RD − 0.001, 95% CI − 0.05, 0.05). However, the results may have been affected by disruption from the COVID-19 pandemic, and unexpectedly high study attrition levels made it impossible to statistically rule out possible moderate-sized effects.

**Supplementary Information:**

The online version contains supplementary material available at 10.1007/s10461-025-04822-8.

## Introduction

The prevalence of HIV and sexually transmitted infections (STIs) is alarmingly high among female sex workers (FSW) in low- and middle-income countries [1–4]. Estimates suggest that FSW have 30 times the odds of HIV acquisition compared to the general female population [5] and 37% of FSW in Sub-Saharan Africa are living with HIV [2]. There is limited access to critical prevention and testing services [6], with nearly two-thirds of all sex workers reporting that they do not know their HIV status [5]. While substantial progress in HIV prevention has been made following the UNAIDS 90-90-90 targets, large gaps remain for FSW, a key population who experiences heightened vulnerability to disease due to high-risk sexual exposures with clients.

Reducing incident HIV and STI infection and increasing access to treatment for FSWs is imperative for improving their health and well-being and decreasing risk of onward transmission to the general population [7]. FSW are a central node of HIV transmission [8], thus investing in HIV prevention in this population can be a cost-effective strategy to lower HIV infection rates globally. UNAIDS calls for evidence-informed responses to increase access and remove structural barriers to HIV prevention and care, including interventions addressing economic insecurity [5]. Poverty is a common driver of sex work and can exacerbate risk of HIV/STIs by creating urgency to work and limiting negotiating power with clients, particularly in the context of increased pay for condomless sex [9].

Financial incentives are a promising approach to incentivize safe sex and do not need to be large to be beneficial, as small sums represent a high proportion of income among individuals experiencing poverty [10]. A growing body of literature suggests that conditional incentive approaches are a promising approach for improving HIV and STI testing and prevention [11, 12]. For example, a study in Malawi found that offering small financial incentives after HIV testing doubled the proportion of individuals who returned to receive their results [13]. In Lesotho, a trial investigating the effect of a lottery intervention on STI infection provided $50 and $100 USD to participants who tested negative for syphilis and trichomoniasis and found a 21.4% decrease in HIV incidence among participants in the lottery groups [14]. In Eswatini, cash transfers conditional on schooling participation combined with lottery incentives conditional on remaining STI negative were found to reduce HIV incidence by 37.8% among adolescent girls and young women, an impact comparable to the most effective universal test and treat interventions [15]. A study in Zimbabwe demonstrated that fixed and lottery-based incentives can increase uptake of HIV testing among older children and young adults [16].

In addition, a previous study in Tanzania, the RESPECT trial (“Rewarding STI Prevention and Control in Tanzania”) examined the effect of two different conditional cash transfer amounts ($10 and $20 USD) on risky sexual behavior. Cash transfers were offered every four months conditional on negative test results for a set of curable STIs. We found that offering cash awards of $20 USD resulted in a significant decrease in STI prevalence after one year, while there was no effect in the $10 USD group [17]. A pilot study was conducted to test this approach among FSW in Tanzania and found that providing cash conditional on negative STI tests was an acceptable and feasible approach in this population, however we were not powered to detect differences in incident infection and did not examine HIV [18]. Building on this evidence, we sought to examine whether conditional financial incentives are effective in reducing risk of HIV and STI infection among a larger sample of FSW. A low-probability, high-reward lottery intervention was developed based on theory positing that individuals often prefer large rewards over small rewards, even if the probability of winning the large reward is small [19–21]. Lottery-based incentives have been shown to be effective among risk-loving individuals in previous trials [14] and are relatively low cost to implement and to scale-up.

The RESPECT 2 trial investigated the effect of lottery-based financial incentive intervention on the combined incidence of HIV and HSV2 among 2,206 FSW in Dar es Salaam, Tanzania over 2018–2022. The objective of our study was to understand whether a low-probability, high-reward incentive scheme offering a 100,000 TZS ($50 USD) reward to randomly selected lottery participants conditional on negative STI results could improve HIV and STI outcomes among FSW.

## Methods

### Study Setting

The trial was conducted in the city of Dar es Salaam, which has the largest population of FSW in Tanzania [18]. The estimated HIV prevalence among females in mainland Tanzania aged 15–64 years is 6.4%, 4.7% among all adults in Dar es Salaam [22]. The latest estimate of HIV prevalence among female sex workers in Dar-es-Salaam dates back from 2010 and was 31.4% [23].

### RESPECT 2 Study Design and Participant Recruitment

Recruitment began August 15, 2018, and concluded on February 1, 2019. The “Rewarding STI Prevention and Control in Tanzania” (RESPECT 2) study was designed as a two-arm parallel randomized controlled trial, with individuals as the unit of randomization. FSW were recruited using respondent-driven sampling (RDS), a chain referral sampling method designed to recruit hard-to-reach and hidden populations. Ten FSW who had previously assisted with intervention studies (and who were not themselves enrolled in our study) were trained in outreach and each recruited five FSW who served as the initial enrollees and as “seeds” for further recruitment. These ten FSW were identified through local health staff working with the FSW community and were selected to represent FSW in areas of Dar es Salaam with high sex work activity. Seeds were then given three coded coupons to recruit their peers into the study from various venues (bars, brothels, street); each participant who successfully enrolled in the study was then herself given three coupons to recruit additional peers into the study, with this process continuing until the desired sample size was achieved. Seeds and participants who helped with subsequent recruitment were given 4,000 TZS (approximately $2 USD) for each of up to 3 eligible FSW that were recruited. Fingerprint scanners were used to prevent multiple registrations across the four study sites, as well as to assist in participant identification verification during subsequent study visits.

Individuals in possession of a coupon who were female, had exchanged sexual intercourse for money in the past six months, were ≥ 18 years of age, HIV-negative, not currently pregnant, had a cellphone able to receive text messages, were able to provide informed consent, and living in Dar es Salaam and planning to remain for at least two years were eligible for inclusion in the trial. Individuals who were HSV2 positive at baseline – but not HIV positive – were also eligible for inclusion since they were still at risk of HIV.

HIV testing was conducted during the enrollment visit at the PASADA study site as part of the eligibility screening for the trial, with positive test results verified with confirmatory testing. Potential participants received a referral paper with their name and unique ID, which was presented to clinicians from PASADA. Clinicians provided HIV counseling, an explanation of the HIV blood test procedure, and a referral to the clinic lab for testing, where a finger prick sample was taken and processed. The results from the blood test were delivered in a private office after verifying the unique ID and name of each individual. Individuals who tested positive were referred to HIV treatment services available at no charge to patients in Tanzania. Those who were determined to be HIV-negative and met all other eligibility criteria were enrolled in the study by research assistants after providing informed consent. Enrolled participants were also tested for HSV2 and syphilis using fingerstick-based rapid blood tests, and for trichomonas vaginalis using a vaginal swab. Syphilis and trichomonas test results were returned within 30 min, and positive syphilis tests were then verified through confirmatory testing; those testing positive for these STIs were offered free treatment.

### Randomization to Control and Treatment Arms

**Fig. 1:**
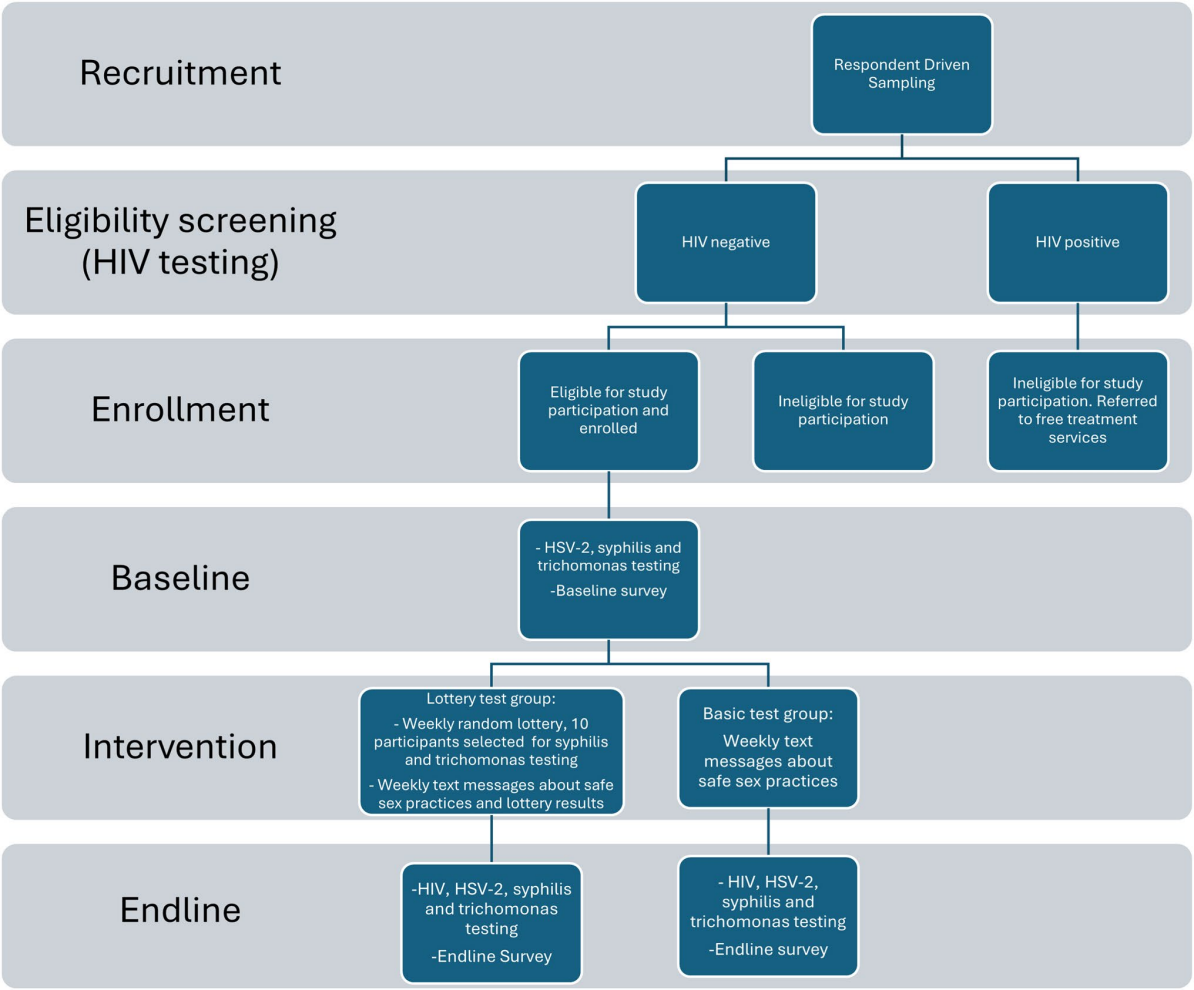
Study procedures

Enrolled participants were individually randomly assigned using opaque envelopes in a 1:1 ratio to the basic test group (control arm), or the lottery test group (intervention arm). The random envelope selection was conducted by the study staff at the end of the enrollment visit (after informed consent, interview completion, counseling, and testing). The main differences between control and intervention arm are described in Table 1 and the study procedures are described in Fig. 1.

**Table 1:** Comparison of interventions in control and treatment arm

Intervention	Basic test group (control arm)	Lottery test group (intervention arm)
No-cost testing and counseling for HIV and STIs at baseline and endline	Yes	Yes
Group counseling session at baseline on negotiating safe sex	Yes	Yes
Biweekly text messages encouraging safe sex practices	Yes	Yes
Random weekly lottery for syphilis and trichomonas testing. An award of 100,000 TZS (~ $50 USD) was offered to ten randomly selected participants each week conditional on negative test results	No	Yes
Weekly messages with the number of participants who won the lottery the previous week	No	Yes

Both groups received baseline and endline no-cost testing and counseling for HIV and STIs. At baseline, all women also participated in an hour-long group counseling session on ways to negotiate safer sexual behaviors to reduce the risk of acquiring STIs. The counseling was conducted by trained clinicians at Pastoral Activities and Services for people with AIDS Dar es Salaam Archdiocese (PASADA).

Both groups also received biweekly text messages encouraging the women to stay healthy. A randomly selected half of the women in each treatment group received the message, “RESPECT 2 update: Do you know how to practice safe sex? Safe sex is protected sex. Use condoms to help reduce the spread of STIs and unplanned pregnancies.” The other half of the women in each treatment group received the message, “RESPECT 2 update: You are still enrolled in the RESPECT 2 study. We encourage you to ‘stay safe’. Women who were concerned about the explicit nature of the first message could opt to receive the latter message instead.

### Intervention

The lottery group was additionally offered entry into a random weekly lottery for syphilis and trichomonas testing. An award of 100,000 TZS (~ $50 USD) was offered to ten randomly selected participants in the lottery group per week, conditional on negative test results. This amount was selected in consultation with local stakeholders as it was seen as scalable and salient enough without exerting undue influence. Participants who had a positive test received free treatment for STIs, additional counseling, and were eligible for future lottery drawings. No masking was used in the study.

On average, each participant in the lottery group could expect to be randomly selected approximately once over the intervention period (104 weeks). The reward of 100,000 TZS for negative STI tests was determined through community discussions and pilot testing to be large enough to incentivize FSW to change their sex work practices and reduce risky sexual behavior and modest enough to prevent any undue coercion.

The lottery group also received a weekly message with the number of women who won the lottery the previous week, to increase the salience of the lottery incentive: “RESPECT 2 update: Last week [X] lucky women won TZS100,000 after testing negative for STIs. You could be the next winner if you remain STI-free. If you have changed number, please respond to this text with your new number.” In order to maintain parity in text message outreach frequency, the control group received at the same time a weekly message but omitting the lottery information: “RESPECT 2 update: You are still enrolled in the RESPECT 2 study. If you have changed number, please respond to this text with your new number.”

Intervention group lotteries began in October 2018, and on average women were eligible for the lottery for 104 weeks (some slightly more or less depending on the date of their recruitment). Study activities were suspended from March to November 2020 due to COVID-19. The final lottery was conducted in May 2021. Endline interviews were conducted between June 2021 and January 2022. Thus, although women were exposed to the equivalent of two years of lottery incentives, these were distributed over a period of more than two and a half years, and the average time between baseline and endline interviews was approximately three years.

### Endline Testing

All non-attriting participants were tested at endline for the same STIs (HIV, HSV2, syphilis, and trichomonas) using the same testing, counseling, and treatment procedures implemented at baseline. The one exception is that participants who tested positive for HSV2 at baseline were not re-tested for HSV2 at endline, since we tested for IgG antibodies that indicate whether the individual has ever been infected with HSV2. In order to verify the accuracy of the HSV2 tests, at endline we compared the results of the OnSite Duo rapid test with an ELISA laboratory test for 30 of the participants, finding 80 percent sensitivity and specificity, which we deemed minimally acceptable.

### Survey Procedures

Baseline and endline surveys were administered in Kiswahili on tablets by research assistants from Innovations for Poverty Action Tanzania. These surveys were conducted in private spaces at the PASADA clinic.

### Participant Tracing

The primary approach used for tracking participants in this unstable FSW study population was via cell phone, with both main and backup numbers requested at enrollment. In late 2019, however, the Tanzanian government required that all cell phones be registered to a national identification card along with biometric confirmation; particularly among those engaged in illegal activity such as FSW, this resulted in many phone numbers being suddenly changed after the government disconnected unregistered phone numbers starting in January 2020. Based on an increase in unsuccessful call attempts, this appeared to significantly contribute to the loss of tracking of our study participants, though we were able to reach a subset of participants at new phone numbers by contacting them through secondary means including via the participant who had originally recruited them. Thus, during the COVID-19 pandemic fieldwork hiatus in mid 2020 we attempted to call all 794 lottery-arm participants who had not yet been called for lottery-based random STI testing. However, we were only able to reach 49 percent of this sample, with 36 percent of the phone numbers permanently disconnected. We similarly attempted to contact a randomly selected 55 percent of the control group and were able to reach 58 percent of them. Others were unable to be reached after three attempts, but this was during a time of extreme pandemic-related disruption; instead of continuing to attempt phone contact at this time, we expanded plans for supplemental endline tracking.

At endline, we again attempted to contact all participants to invite them for endline surveys and HIV/STI testing. Research staff first called the current primary phone number of each participant. Three attempts at different times of day were made for each participant. If all attempts on the primary phone were unsuccessful, all alternate phone numbers were attempted. Pamphlets were also distributed at common work sites such as brothels and bars, and in-person tracing was conducted by RDS seeds and study staff at 25 FSW business centers and sex work locations (bars, brothels, guest houses) to attempt to locate participants unreachable by phone. An endline lottery was introduced in September 2021 to incentivize participation, as a large proportion of participants had not completed surveys or testing: one of every five endline participants each week was selected to win 50,000 TZS for participating. Those who participated were also asked to locate any peers or colleagues enrolled in the study and bring them to PASADA to complete their survey and testing; one of every ten participants who recruited others in this way was selected to win 50,000 TZS. After September 2021, the endline lottery was replaced by a uniform incentive amount of 10,000 TZS to all participants who completed endline surveys.

### Outcomes

The primary outcome was combined incidence of HIV and/or HSV2, at 36 months after baseline (pre-specified for 24 months, but extended due to the COVID-19 disruption). The combined outcome of HIV and/or HSV2 was selected to increase statistical power, given the low incidence of HIV alone in the study population. Both HIV and HSV-2 are incurable STIs that share the same underling behavioral risk factors, making their combined incidence an epidemiologically meaningful composite outcome in the context of STI prevention.

Prespecified secondary outcomes included the prevalence of syphilis and trichomonas vaginalis, two curable sexually transmitted infections, sexual behavior, and incident HIV and HSV2 infection (separately). Participants were coded as having incident HSV2 only if they tested negative for HSV2 at baseline and positive at endline. The outcomes of HIV, HSV2, syphilis and trichomonas vaginalis were measured through testing. Sexual behavior was self-reported in surveys.

### Sample Size

The RESPECT 2 trial was powered based on the primary outcome of combined incidence of HSV2 and/or HIV. Assuming 15% combined incidence in the control group over a two-year trial period and a target minimum detectable effect of 4.5 percentage points (a 30 percent risk reduction), to achieve 80% power we aimed to enroll at least 1,078 participants per study arm, accounting for 20% attrition over the study period (for a minimum of 2,156 participants overall).

### Statistical Analysis

Data analysis was conducted in STATA version 15.1 [24]. We first descriptively explored demographic characteristics of participants and self-reported sexual behavior and sexual health history, including number and type of clients, income, condom use, prevalence of STIs, and previous STI and HIV testing. Characteristics between treatment groups and baseline and endline participants were compared.

The primary intent-to-treat analysis included only participants who completed the study, as the primary outcome of combined HIV/HSV2 incidence was measured at endline. Linear probability models were used to estimate unadjusted and regression adjusted risk differences (RD) with 95% confidence intervals (CI). Control variables in the adjusted models were baseline: age, education, amount of time living in Dar es Salaam, marital status, number of children, total monthly income, monthly income from sex work, social ladder ranking, location of sex work, number of clients, price received for sex with a condom, previous STI diagnosis, timing of last STI testing, preferred frequency of STI testing, previous HIV testing, perceived HIV risk, and baseline value for the dependent variable being analyzed.

In sensitivity analyses, we also report results of the adjusted models for the primary outcome after applying inverse probability of censoring weighting (IPCW) to account for systematic observed differences in participants who were lost to follow-up. The probability of participating in endline (not attriting) was generated using logistic regression as a function of the same baseline control variables listed above. Weights were calculated using the predicted probability of not attriting and were included in adjusted analyses.

Finally, for our main endpoint (combined HIV/HSV2 incidence), we also estimate unadjusted treatment effect bounds following Lee [25], using the *leebounds* command in STATA. We report these upper and lower bound treatment effect estimates which vary the assumptions regarding nonrandom selection of the FSW who attrited from the endline survey. This is a non-parametric bounding that drops a set of the Treatment arm observations to match the proportion of Control arm observations not attriting. The upper (lower) bounds are computed by dropping these Treatment arm observations from the lower (upper) end of the distribution of the dependent variable and re-estimating the unadjusted treatment effects model.

### Ethics

The RESPECT 2 trial received ethical approval from the Committee for Protection of Human Subjects at University of California, Berkeley (Protocol 2015-08-7849) and the National Institute for Medical Research in Tanzania (NIMR/HQ/R.8a/Yol.IXI 2770). All data collection was carried out by research assistants from Innovations for Poverty Action Tanzania who were trained in ethical research practices. Written informed consent was obtained from all participants, other than those who were illiterate and provided verbal consent.

## Results

**Fig. 2:**
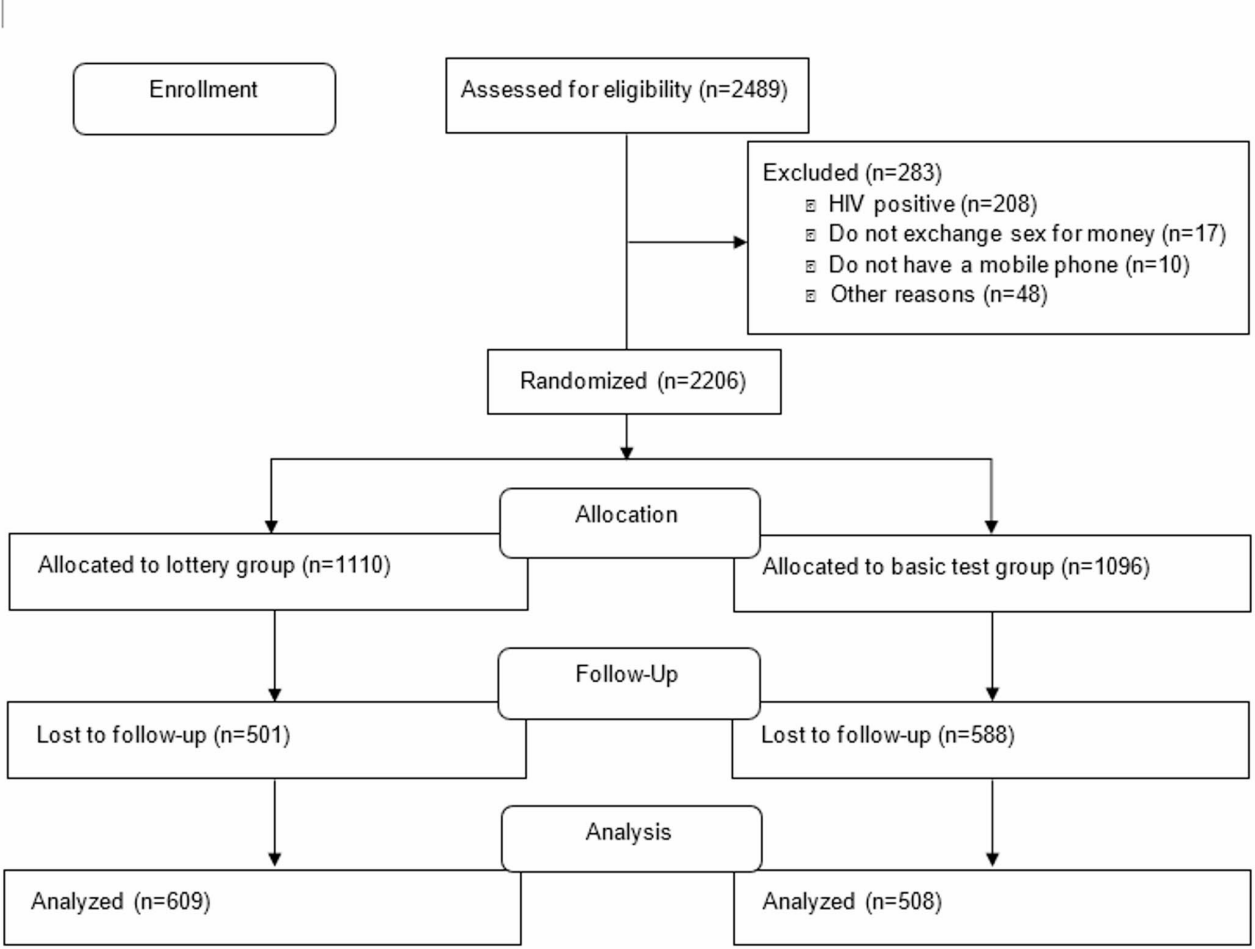
Trial profile diagram

From August 15, 2018, to February 1, 2019, 2,489 individuals with valid RDS coupons were screened for eligibility at a PASADA study site (Fig. 2). Of those, 283 did not meet the eligibility criteria and were excluded; the remaining 2,206 were enrolled in the trial and randomized (1,110 allocated to the lottery group, 1,096 allocated to the basic test group). This enrollment size slightly exceeded our target sample size based on the above power calculations. Participants were followed for approximately 36 months, in order to achieve 24 months of incentive intervention exposure after accounting for the COVID-related study disruption. At endline, 1,089 (49.4%) were not able to be reached by phone or in-person tracing and were determined to be lost to follow-up. Attrition was significantly higher in the basic test group than in the lottery group (53.6% vs. 45.1%, Online Appendix Table 1).

Across the 104 intervention weeks, 63% of the lottery group participants whose names were drawn for random testing responded to the invitation and went during that week to the PASADA study site for syphilis and trichomonas testing. Appendix Fig. 1 shows the weekly proportion reporting for testing (among the 10 randomly selected each week). Those who did not report for testing were sometimes traveling out of town, but more commonly were not able to be reached (consistent with the above-discussed loss-to-follow-up). Among those who did report for testing during those 104 weeks, 98% tested negative and thus received the Tsh100,000 incentive reward, and the other 2% tested positive and thus were offered free treatment (with no reward). Appendix Fig. 2 shows the proportion testing positive each week.

Demographic characteristics of study participants were balanced across treatment arms at baseline (Table 2). The mean age of participants was 26 years; only 10.7% had not completed primary school, and 58.9% had completed primary school as their highest level of education. Most participants were never married (76.7%), with 20.1% divorced, and 70.6% had at least one child. The mean monthly income from sex work was 236,869 TZS (~ $100 USD/month). The final analysis sample was statistically significantly older in age and had a higher proportion of FSW who were never married, divorced, had at least one child, and had lived in Dar es Salaam their whole lives compared to those who were lost to follow-up (Online Appendix Table 2), although these differences were not large. In Online Appendix Table 3 we further explore attrition by comparing baseline characteristics of the endline sample split between those who we were able to reach in the initial phase of endline outreach (through June–July 2021), versus those who were interviewed at endline only after more intensive outreach (August 2021–January 2022). There were statistically significant differences between early versus late responders in age, education, having at least one child, and place of sex work, but overall, those differences were minor, again providing little evidence of systematic attrition. Similarly, there were no meaningful differences in baseline characteristics in the final sample by treatment arm (Online Appendix Table 4).

**Table 2:** Baseline demographic characteristics by treatment arm

	Treatment (n = 1110)		Control (n = 1096)			Overall (n = 2206)	
	Mean	SD	Mean	SD	p-value	Mean	SD
Respondent age	26.568	6.814	26.424	6.627	0.614	26.497	6.720
Education							
No formal education	0.038	0.191	0.035	0.183	0.691	0.036	0.187
Some primary complete	0.069	0.254	0.063	0.243	0.545	0.066	0.249
Primary complete	0.584	0.493	0.595	0.491	0.596	0.589	0.492
Some secondary complete	0.123	0.329	0.132	0.339	0.533	0.128	0.334
Secondary complete	0.174	0.379	0.169	0.375	0.752	0.171	0.377
High school	0.003	0.052	0.002	0.043	0.665	0.002	0.048
Vocational	0.005	0.073	0.000	0.000	0.015	0.003	0.052
University	0.004	0.060	0.005	0.067	0.724	0.004	0.064
Lived in Dar es Salaam all their life	0.708	0.455	0.711	0.453	0.864	0.710	0.454
Marital status							
Never married	0.766	0.424	0.750	0.433	0.388	0.758	0.428
Divorced/separated	0.201	0.401	0.212	0.409	0.532	0.206	0.405
Currently married	0.014	0.116	0.016	0.124	0.695	0.014	0.116
Widowed	0.009	0.095	0.018	0.134	0.061	0.014	0.116
Cohabiting	0.010	0.099	0.005	0.067	0.139	0.007	0.085
Has at least one child	0.706	0.456	0.706	0.456	0.996	0.706	0.456
Neighborhood poverty ranking (1–5)	2.678	0.702	2.691	0.665	0.658	2.684	0.684
Self-rated social status (1–7)	3.717	1.522	3.719	1.534	0.974	3.718	1.528
Income							
Total income (30 days) TZS	257,409	303,439	251,222	346,703	0.661	254,325.220	325,657.824
Income from sex work (30 days) TZS	244,734	304,488	229,004	195,184	0.153	236,854.655	255,706.474
Place of work							
Pub/bar	0.411	0.492	0.412	0.492	0.945	0.411	0.492
Guesthouse	0.257	0.437	0.239	0.427	0.322	0.248	0.432
Street	0.085	0.279	0.099	0.299	0.253	0.092	0.289
Night club/disco	0.079	0.270	0.080	0.271	0.920	0.079	0.271
Brothel	0.069	0.253	0.067	0.250	0.871	0.068	0.252
Clients and condom use							
Number of clients/week	8.342	6.460	8.493	7.143	0.606	8.364	6.587
Amount earned with a condom	13,280	14,287	13,375	17,015	0.890	13,327.408	15,692.265
Amount earned without a condom	18,133	31,327	18,270	39,207	0.936	18,202.245	35,518.773
Ever had an STI	0.077	0.267	0.064	0.245	0.219	0.071	0.257
Last time tested							
Within the last month	0.017	0.130	0.022	0.146	0.417	0.019	0.138
1–2 months ago	0.100	0.300	0.086	0.280	0.250	0.093	0.290
3–6 months ago	0.162	0.369	0.169	0.375	0.675	0.165	0.372
6 months—1 year ago	0.113	0.316	0.121	0.327	0.523	0.117	0.321
Over a year ago	0.188	0.391	0.177	0.382	0.493	0.183	0.386
Never	0.420	0.494	0.425	0.495	0.799	0.422	0.494
Reasons for not STI testing							
Fear of knowing status	0.318	0.466	0.350	0.477	0.298	0.334	0.472
Don’t feel at risk	0.120	0.326	0.086	0.280	0.085	0.135	0.342
Not important to me	0.120	0.326	0.086	0.280	0.085	0.103	0.304
Cost	0.071	0.257	0.058	0.234	0.424	0.064	0.246
Didn’t know where to go	0.062	0.242	0.056	0.230	0.677	0.059	0.236
Concerned about confidentiality	0.041	0.198	0.062	0.242	0.139	0.052	0.221
Negative attitude of healthcare worker	0.047	0.212	0.039	0.193	0.518	0.043	0.203
Perceived risk for HIV							
High risk	0.590	0.492	0.601	0.490	0.593	0.596	0.491
Medium risk	0.168	0.374	0.173	0.379	0.718	0.170	0.376
Low risk	0.101	0.301	0.068	0.253	0.006	0.085	0.279
Not at risk	0.123	0.328	0.135	0.342	0.380	0.129	0.335
Ever been tested for HIV	0.920	0.272	0.897	0.304	0.062	0.908	0.288

*SD* standard deviation

At baseline, the prevalence of HSV2 was 29.8% (29.13% control versus 30.51% lottery arm), the prevalence of syphilis was 2.6% (2.08% control versus 3.12% lottery arm) and trichomoniasis vaginalis was 2.8% (2.77% control versus 3.22% lottery arm). There were no statistically significant differences in these baseline STI rates by treatment arm. All participants were HIV-negative, as indicated by the inclusion criteria.

For the main endpoint as measured at 36 months, the control arm had a combined HIV/HSV2 incidence at endline of 17.3%, similar to the 15% that was assumed in the ex-ante power calculations. The lottery arm had only a slightly lower combined HIV/HSV2 incidence at endline, 16.8%, resulting in an unadjusted difference between treatment arms that was not statistically significant at the 5% level, indicating no effect of the lottery intervention on the primary outcome (RD: − 0.006, 95% CI − 0.05, 0.04) (Table 3). The adjusted risk difference and IPCW weighted models also indicated null results, with identical findings (RD: 0.001, 95% CI − 0.05, 0.05). The study had been powered for a minimum detectable effect size of a 4.5 percentage point reduction in incidence; the higher than expected attrition level resulted in the lower bound of the confidence interval being able to reject an effect size of greater than 5 percentage points. At the actual control arm incidence of 17.3%, we note that the estimated confidence interval can still reject a 29% risk reduction, similar to the 30% risk reduction on which the power calculation was based.

**Table 3:** Effect of lottery intervention on primary and secondary outcomes at 36 months

	Control (n = 508)	Lottery (n = 609)	Unadjusted RD (95% CI)	Adjusted RD (95% CI)	Weighted RD (95% CI)
HIV/HSV2 incidence*^†^	84 (17.3%)	98 (16.8%)	− 0.006 (− 0.05, 0.04)	0.001 (− 0.05, 0.05)	0.001 (− 0.05, 0.05)
HIV incidence	9 (1.8%)	8 (1.3%)	− 0.005 (− 0.02, 0.01)	− 0.007 (− 0.03, 0.01)	− 0.007 (− 0.04, 0.01)
HSV2 incidence**	78 (24.5%)	92 (25.8%)	0.012 (− 0.05, 0.08)	0.021 (− 0.05, 0.09)	0.018 (− 0.05, 0.09)
HSV2 endline prevalence	240 (47.24%)	300 (49.26%)	0.020 (− 0.04, 0.08)	0.022 (− 0.03, 0.08)	0.022 (− 0.03, 0.08)
Syphilis prevalence	13 (2.6%)	21 (3.4%)	0.009 (− 0.01. 0.03)	0.003 (− 0.01, 0.02)	0.002 (− 0.01, 0.02)
Trichomoniasis vaginalis prevalence	1 (0.2%)	2 (0.3%)	0.001 (− 0.00, 0.01)	− 0.002 (− 0.01, 0.01)	0.002 (− 0.01, 0.01)

Data presented as frequency and %

Risk differences (RD) with 95% CI generated using linear probability models with robust standard errors

Adjusted and weighted models controlled for baseline age, education, amount of time living in Dar es Salaam, marital status, children, total monthly income, monthly income from sex work, social ladder ranking, location of sex work, number of clients, price received for sex with a condom, previous STI testing, last time of STI testing, frequency of STI testing, previous HIV testing, perceived HIV risk, and baseline test result

*Primary outcome; combined incidence of HIV and HSV2, including baseline HSV2 positives among non-incident cases

**HSV2 incidence estimated only among participants who tested negative for HSV2 at baseline (control n = 318; lottery n = 357)

^†^Missing data: HIV/HSV2 incidence (n = 49), HSV2 incidence (n = 317), Trichomoniasis (n = 44)

We attempted to further explore the effects of attrition through estimating Lee bounds on the treatment effect in the unadjusted model for our main endpoint of combined HIV/HVS2. The Lee lower bound was − 0.1650, indicating that depending on the degree of differential attrition there was the possibility of a much larger beneficial effect of the lottery, although the 95 percent confidence interval on the lower bound of [− 0.2643, − 0.0657] is quite wide. The Lee upper bound was 0.0264, which as a point estimate indicates little potential harm, but again the wide confidence interval of [− 0.0260, 0.0788] makes this less informative.

There were also no significant differences observed at endline between treatment arms when assessing HIV and HSV2 infection independently. Incident HIV infection was 0.5 percentage points lower in the lottery arm than in the control arm (1.8% control vs. 1.3% lottery arm), while incident HSV2 infection (excluding those who tested HSV2 positive at baseline) was 1.2 percentage points higher in the lottery arm (24.5% control vs. 25.8% lottery arm). Compared to control, the lottery arm had a higher prevalence of syphilis (2.6% control vs. 3.4% lottery arm) and trichomoniasis vaginalis (0.2% control and 0.3% lottery arm) at endline, but again these differences were not statistically significant.

Self-reported sexual behaviors at endline were also similar across treatment arms. Point estimates indicate that some behaviors were self-reported as less risky among the lottery arm versus the control arm, however these differences were not statistically significant at the 5% level; e.g., participants in the lottery arm were more likely than the control arm to self-report that they decreased the frequency of exchanging sex for money (55.3% vs. 50.0%) (Table 4).

**Table 4:** Self-reported sexual behaviors and influencing factors at 36 months

	Control (n = 508)	Lottery (n = 609)	Unadjusted RD (95% CI)	Adjusted RD (95% CI)	Weighted RD (95% CI)
Number of clients	6.199	6.253	0.054 (− 0.65, 0.76)	0.219 (− 0.52, 0.95)	0.250 (− 0.51, 1.01)
Perceived low/no risk for HIV*	146 (29.0%)	187 (30.9%)	0.019 (− 0.04, 0.07)	0.014 (− 0.05, 0.07)	0.0129 (− 0.05, 0.07)
Decreased frequency of exchanging sex for money	254 (50.0%)	337 (55.3%)	0.053 (− 0.01, 0.11)	0.049 (− 0.02, 0.11)	0.0487 (− 0.018, 0.12)
Less sexual partners	278 (54.7%)	336 (55.1%)	0.004 (− 0.05, 0.06)	− 0.004 (− 0.07, 0.06)	0.001 (− 0.07, 0.07)
More use of condoms during sex	248 (48.8%)	293 (48.1%)	− 0.007 (− 0.07, 0.05)	0.016 (− 0.08, 0.05)	− 0.007 (− 0.07, 0.06)
Less anal sex	200 (39.4%)	260 (42.7%)	0.033 (− 0.25, 0.09)	0.000 (− 0.06, 0.06)	0.004 (− 0.06, 0.07)
Number of sexual partners used a condom with in the last week	3.9 (4.4)	4.3 (4.98)	0.349 (− 0.24, 0.94)	0.271 (− 0.27, 0.91)	0.371 (0.27, 1.01)
Amount received for last sex with a condom (TSH)	15,493.0	16,462.9	969.830 (− 1065.73, 3005.39)	691.461 (− 1456.69, 2839.62)	1011.387 (− 1023.86, 3046.64)
Amount received for last sex without a condom (TSH)	19,278.9	19,749.0	470.125 (− 2509.35, 3449.60)	885.892 (− 2286.14, 4057.93)	1319.730 (− 1534.05, 4173.51)

Data presented as frequency and %

Risk differences (RD) with 95% CI generated using linear probability models with robust standard errors

Adjusted and weighted models controlled for baseline age, education, amount of time living in Dar es Salaam, marital status, children, total monthly income, monthly income from sex work, social ladder ranking, location of sex work, number of clients, price received for sex with a condom, previous STI testing, last time of STI testing, frequency of STI testing, previous HIV testing, perceived HIV risk

*Missing data: Number of clients (adjusted n = 135, weighted n = 137), perceived low/no risk for HIV (unadjusted n = 7, adjusted n = 142, weighted n = 144), decreased r frequency of exchanging sex for money (adjusted n = 135, weighted n = 137), less sexual partners (adjusted n = 135, weighted n = 137), more use of condoms during sex (adjusted n = 135, weighted n = 137), less anal sex (adjusted n = 135, weighted n = 137), number of sexual partners used a condom with in the last week (unadjusted n = 63, adjusted n = 75, weighted n = 103), amount received for last sex with a condom (unadjusted n = 75, adjusted n = 184, weighted n = 185), amount received for last sex without a condom (unadjusted n = 103, adjusted n = 209, weighted n = 210)

Agreement was high among participants in both arms that the STI testing, free STI treatment, counseling during study enrollment, and regular text messages with information about safe sex they received during the intervention period contributed to reducing their risky sexual behaviors (Table 5). Of these endline respondents, 98% indicated that they would participate in a similar program in the future.

**Table 5:** Perceptions of the RESPECT 2 intervention and previous experiences with STI/HIV testing and diagnosis

	Control (n = 508)	Lottery (n = 609)	Unadjusted RD (95% CI)	Adjusted RD (95% CI)	Weighted RD (95% CI)
Perceptions of RESPECT 2 intervention					
STI testing contributed to reducing risky sexual behavior	394 (77.6%)	488 (80.1%)	0.026 (− 0.02, 0.07)	0.012 (− 0.04, 0.07)	0.021 (− 0.03, 0.08)
Free STI treatment contributed to reducing risky sexual behavior	333 (65.6%)	427 (70.2%)	0.046 (− 0.01, 0.10)	0.029 (− 0.03, 0.09)	0.028 (− 0.04, 0.09)
Counseling at enrollment contributed to reducing risky sexual behavior	402 (79.1%)	506 (83.1%)	0.040 (− 0.01, 0.09)	0.052 (0.00, 0.10)	0.057 (0.01, 0.11)
Text messages contributed to reducing risky sexual behavior	363 (71.5%)	460 (75.6%)	0.041 (− 0.11, 0.09)	0.047 (− 0.01, 0.10)	0.056 (0.00, 0.12)
Enrollment contributed to abstaining more from sex	282 (55.5%)	352 (57.8%)	0.023 (− 0.04, 0.08)	0.035 (− 0.03, 0.10)	0.032 (− 0.03, 0.10)
Enrollment contributed to choosing less risky sexual partners	358 (70.5%)	417 (68.5%)	− 0.020 (− 0.07, 0.03)	− 0.020 (− 0.80, 0.04)	0.022 (− 0.08, 0.04)
Participation in other STI programs					
Agree to participate in program with STI testing awards for negative results	495 (97.8%)	598 (98.7%)	0.009 (− 0.01, 0.02)	0.010 (− 0.01, 0.03)	0.010 (− 0.01, 0.03)
Participated in another program with syphilis testing	70 (13.8%)	83 (13.6%)	− 0.002 (− 0.04, 0.04)	− 0.013 (− 0.06, 0.03)	− 0.015 (− 0.06, 0.03)
Participated in another program with trichomoniasis testing	70 (13.8%)	83 (13.6%)	− 0.002 (− 0.04, 0.04)	− 0.013 (− 0.06, 0.03)	− 0.015 (− 0.06, 0.03)
Participated in another program with other STI testing	119 (23.4%)	138 (22.7%)	− 0.010 (− 0.06, 0.04)	− 0.027 (− 0.08, 0.03)	− 0.030 (− 0.09, 0.03)
Participated in another program with STI treatment	18 (3.5%)	31 (5.1%)	0.015 (− 0.01, 0.04)	0.008 (− 0.02, 0.03)	0.012 (− 0.01, 0.04)
Participated in another program with condom distribution	98 (19.3%)	127 (20.9%)	0.016 (− 0.03, 0.06)	0.017 (− 0.03, 0.07)	0.009 (− 0.04, 0.06)
Participated in another program with counseling on STIs and safe sex	155 (30.5%)	179 (29.4%)	− 0.011 (− 0.07, 0.04)	− 0.016 (0.08, 0.04)	− 0.020 (0.08, 0.04)
Other STI/HIV diagnosis and testing					
Diagnosed with STI outside of study	13 (2.6%)	14 (2.3%)	− 0.003 (− 0.02, 0.02)	− 0.005 (− 0.03, 0.02)	− 0.002 (− 0.02, 0.02)
Never tested for STIs other than this study	333 (65.6%)	397 (65.2%)	− 0.003 (− 0.06, 0.05)	0.035 (0.03, 0.10)	0.042 (− 0.02, 0.11)
Been tested for HIV outside of this study	338 (76.5%	511 (74.6%)	− 0.019 (− 0.07, 0.04)	− 0.029 (− 0.09, 0.03)	− 0.031 (− 0.09, 0.03)

Data presented as frequency and % or mean (SD)

Risk differences (RD) with 95% CI generated using linear probability models with robust standard errors

Adjusted and weighted models controlled for baseline age, education, amount of time living in Dar es Salaam, marital status, children, total monthly income, monthly income from sex work, social ladder ranking, location of sex work, number of clients, price received for sex with a condom, previous STI testing, last time of STI testing, frequency of STI testing, previous HIV testing, perceived HIV risk

*Missing data: STI testing contributed to reducing risky sexual behavior (adjusted n = 135, weighted n = 137); Free STI treatment contributed to reducing risky sexual behavior (adjusted n = 135, weighted n = 137); Counseling at enrollment contributed to reducing risky sexual behavior (adjusted n = 135, weighted n = 137); Text messages contributed to reducing risky sexual behavior (adjusted n = 135, weighted n = 137); Enrollment contributed to abstaining more from sex (adjusted n = 135, weighted n = 137); Enrollment contributed to choosing less risky sexual partners (adjusted n = 135, weighted n = 137); Agree to participate in program with STI testing awards for negative results (unadjusted n = 5, adjusted n = 138, weighted n = 140), Participated in another program with syphilis testing (adjusted n = 135, weighted n = 137); Participated in another program with trichomoniasis testing (adjusted n = 135, weighted n = 137); Participated in another program with other STI testing (adjusted n = 135, weighted n = 137); Participated in another program with other STI treatment (adjusted n = 135, weighted n = 137); Participated in another program with condom distribution (adjusted n = 135, weighted n = 137); Participated in another program with counseling on STIs and safe sex (adjusted n = 135, weighted n = 137); Diagnosed with STI outside of study (adjusted n = 135, weighted n = 137); never tested for STIs other than this study (adjusted n = 135, weighted n = 137); Been tested for HIV outside of this study (unadjusted n = 164, adjusted n = 227, weighted n = 279)

**Fig. 3:**
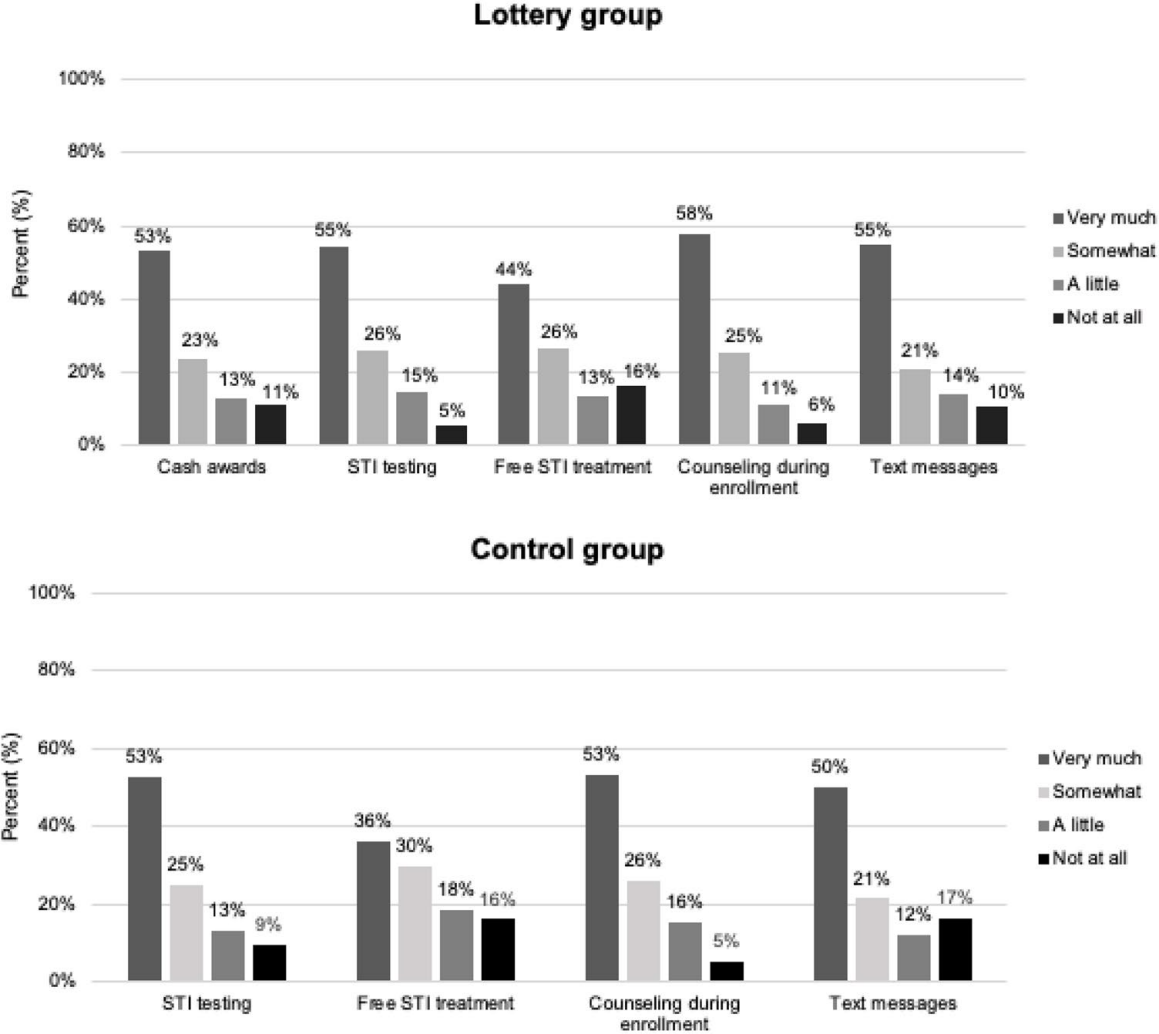
Perceived effect of the intervention on reduced risky sexual behaviors

Despite no significant differences in HIV or STI outcomes or self-reported sexual behaviors between treatment arms, the lottery intervention was viewed favorably by participants in the lottery arm and lottery participants perceived the intervention to reduce their risk behaviors, with 465 (76.4%) agreeing that receiving cash awards for negative test results contributed to reducing their risky sexual behaviors “very much” or “somewhat” (Fig. 3).

## Discussion

The RESPECT 2 trial investigated the effect of a lottery-based incentive on combined HIV/HSV2 incidence at 36 months. Our findings revealed no statistically significant difference in this primary outcome between treatment arms at the 5% level. The confidence intervals can reject a 29% risk reduction for the primary outcome, as compared to the 30% minimum detectable risk reduction that the study was ex-ante powered to detect. We also found no statistically significant differences between the arms in self-reported risky sexual behaviors among FSW. Nevertheless, we found that a large majority of FSW self-reported that STI testing, free treatment, counseling, and text messages with information on safe sex (services offered to all participants in the RESPECT 2 trial) contributed to a reduction in risky sexual behaviors. While we cannot infer from our study design the extent to which these services may positively influence sexual behavior, there clearly is demand for them among this FSW population.

Previous studies have examined the effects of lottery-based incentives on STI and HIV outcomes in the general population [11], however, to our knowledge, this is the first study to assess a lottery intervention among the FSW population. While lotteries have been found to have positive impacts on HIV testing [16], reduced HIV incidence [14], and time to ART initation [26] in some settings in Sub-Saharan Africa, other findings demonstrate that lottery incentives have limited effects on these outcomes when provided without a complementary behavioral intervention [15, 27], consistent with what was found in this study.

This study had several limitations though which must be kept in mind when drawing conclusions from our results. First, the 49% attrition rate at endline was unusually high and concerning relative to the 20% attrition assumed in the ex-ante power calculations. The achieved sample size did still result in confidence intervals that just excluded the ex-ante chosen minimum detectable effect of a 30% risk reduction, thus the unadjusted analysis can still be interpreted as finding a null effect of the intervention. An accompanying concern with high attrition levels though is the potential for systematic differences across arms in the characteristics of attriters, which could threaten internal validity. This concern is softened somewhat by the fact that the IPCW model estimates (accounting for differences in observable attrition correlates) were very similar to the unadjusted models. However, the high attrition levels resulted in Lee bounds that were much wider and could not rule out substantial beneficial effects of the intervention.

A second limitation of the study was the unexpected shocks that occurred during the study. New government regulations on cellphone registrations contributed to the unexpectedly high attrition, but also highly consequential was the onset of the COVID-19 pandemic in the second year of the study. The pandemic disruptions changed many aspects of FSW work [28], contributing to an at least temporary decrease in work by most of the FSW, and also likely exacerbated attrition rates. A particularly unexpected result in the endline data is the very low HIV incidence rate, with only 1.5% of the FSW sero-converting during the three-year study period; low HIV incidence is of course good news but raises an external validity question of whether those sero-converting may have been more likely to attrit.

A more substantive consideration in interpreting our results is whether the incentive design may have been insufficient to substantially lower risky behavior. Community collaborators cautioned against setting a lottery incentive of more than Tsh 100,000 (~ US$50), and budget impact analyses may also limit the possibility of scaling incentives larger than this. But with only 10 of 1,110 lottery arm women being drawn each week, each woman only had about 1% chance of being selected for random testing in a given week, thus the weekly expected value of the incentive was only about Tsh 1,000 per week (or an expected value of roughly Tsh 100,000 across the 104 weeks of the intervention). This incentive amount may be too small relative to the potential income loss from reducing risky behaviors (the median price for a client without a condom was Tsh15,000, and the median price with a condom was Tsh 10,000, with mean monthly sex work income at baseline of about Tsh 235,000).

Future studies will be challenged to develop feasible and salient incentive strategies for reducing risky FSW behaviors, as well as in recruiting an even larger sample than the already large sample size of over 2,000 FSW that we enrolled. The FSW who remained in the study reported favorable views of the study—even those in the control group. Although these responses could reflect social desirability bias, their decision to remain in the study suggests that they did find value in the services offered them. How best to leverage this interest with other behavior change intervention designs remains a high priority research question for slowing HIV/STI transmission among this key node for disease spread.

## Supplementary Information

Below is the link to the electronic supplementary material.

Supplementary file1 (DOCX 2130 KB)

## Data Availability

De-identified participant data used in these analyses will be made available upon request.
