# Supplementary material for: Effects of a Lottery Incentive on STI/HIV Incidence Among Female Sex Workers in Tanzania: Outcomes of Rewarding STI Prevention and Control in Tanzania (RESPECT-2)
Source: AIDS Behav. 2025 Sep 1;29(12):3852–65. doi: 10.1007/s10461-025-04822-8 (PMC12580420; doi:10.1007/s10461-025-04822-8)
Supplement: Supplementary file 1 — Supplementary file1 (DOCX 2130 KB) [file 10461_2025_4822_MOESM1_ESM.docx]

**ONLINE APPENDIX TABLES AND FIGURES**

**Supplemental Table 1. Attrition by treatment arm**

|  | **Lottery** | **Control** | **Unadjusted RD**  **(95% CI)** | **Adjusted RD**  **(95% CI)** |
| --- | --- | --- | --- | --- |
| **Lost to follow-up** | 45.1% | 53.6% | -0.09  (-0.13, -0.04) | -0.08  (-0.12, -0.04) |
| Risk differences with 95% CI generated using linear probability models with robust standard errors  Adjusted model controlled for baseline age, education, amount of time living in Dar es Salaam, marital status, children, total monthly income, monthly income from sex work, social ladder ranking, location of sex work, number of clients, price received for sex with a condom, previous STI testing, last time of STI testing, frequency of STI testing, previous HIV testing, perceived HIV risk | | | | |

**Supplemental Table 2. Baseline demographic characteristics, endline sample vs. participants lost to follow-up**

|  | **Endline sample**  **(n=1117)** | |  | **Lost to follow-up**  **(n=1089)** | |  | **p-value** |
| --- | --- | --- | --- | --- | --- | --- | --- |
|  | Mean | SD |  | Mean | SD |  |  |
| **Respondent age** | 27.577 | 7.104 |  | 25.388 | 6.110 |  | 0.000 |
| **Education** |  |  |  |  |  |  |  |
| No formal education | 0.040 | 0.197 |  | 0.032 | 0.176 |  | 0.306 |
| Some primary complete | 0.069 | 0.253 |  | 0.063 | 0.244 |  | 0.599 |
| Primary complete | 0.593 | 0.492 |  | 0.586 | 0.493 |  | 0.746 |
| Some secondary complete | 0.126 | 0.332 |  | 0.129 | 0.336 |  | 0.820 |
| Secondary complete | 0.163 | 0.369 |  | 0.180 | 0.384 |  | 0.288 |
| High school | 0.002 | 0.042 |  | 0.003 | 0.052 |  | 0.634 |
| Vocational | 0.003 | 0.052 |  | 0.003 | 0.052 |  | 0.975 |
| University | 0.004 | 0.067 |  | 0.004 | 0.061 |  | 0.767 |
| **Lived in Dar es Salaam all their life** | 0.750 | 0.433 |  | 0.669 | 0.471 |  | 0.000 |
| **Marital status** |  |  |  |  |  |  |  |
| Never married | 0.721 | 0.449 |  | 0.796 | 0.403 |  | 0.000 |
| Divorced/separated | 0.243 | 0.429 |  | 0.169 | 0.375 |  | 0.000 |
| Currently married | 0.013 | 0.115 |  | 0.016 | 0.124 |  | 0.668 |
| Widowed | 0.013 | 0.111 |  | 0.015 | 0.120 |  | 0.662 |
| Cohabiting | 0.010 | 0.099 |  | 0.005 | 0.068 |  | 0.146 |
| **Has at least one child** | 0.744 | 0.437 |  | 0.668 | 0.471 |  | 0.000 |
| **Neighborhood poverty ranking (1-5)** | 2.682 | 0.684 |  | 2.687 | 0.684 |  | 0.872 |
| **Self-ranked social status (1-7)** | 3.720 | 1.526 |  | 3.715 | 1.530 |  | 0.931 |
| **Income** |  |  |  |  |  |  |  |
| Total income (30 days) Tsh - mean | 256200 | 303441 |  | 252414 | 346987 |  | 0.788 |
| Income from sex work (30 days) Tsh - mean | 238419 | 280312 |  | 235261 | 228049 |  | 0.774 |
| **Place of work** |  |  |  |  |  |  |  |
| Pub/bar | 0.405 | 0.491 |  | 0.419 | 0.494 |  | 0.505 |
| Guesthouse | 0.259 | 0.439 |  | 0.237 | 0.425 |  | 0.225 |
| Street | 0.078 | 0.269 |  | 0.106 | 0.309 |  | 0.023 |
| Night club/disco | 0.087 | 0.283 |  | 0.071 | 0.257 |  | 0.164 |
| Brothel | 0.069 | 0.254 |  | 0.067 | 0.250 |  | 0.802 |
| **Clients and condom use** |  |  |  |  |  |  |  |
| Number of clients /week | 8.372 | 6.861 |  | 8.463 | 6.753 |  | 0.755 |
| Amount earned with a condom | 13942 | 15091 |  | 12700 | 16267 |  | 0.071 |
| Amount earned without a condom | 19731 | 41189 |  | 16697 | 28809 |  | 0.075 |
| **Ever had an STI** | 0.065 | 0.247 |  | 0.076 | 0.266 |  | 0.323 |
| **Last time tested for STIs** |  |  |  |  |  |  |  |
| Within the last month | 0.023 | 0.151 |  | 0.016 | 0.124 |  | 0.193 |
| 1-2 months ago | 0.089 | 0.284 |  | 0.097 | 0.297 |  | 0.482 |
| 3-6 months ago | 0.164 | 0.370 |  | 0.167 | 0.373 |  | 0.835 |
| 6 months - 1 year ago | 0.124 | 0.330 |  | 0.109 | 0.312 |  | 0.268 |
| Over a year ago | 0.194 | 0.396 |  | 0.171 | 0.377 |  | 0.154 |
| Never | 0.406 | 0.491 |  | 0.440 | 0.497 |  | 0.103 |
| **Reasons for not testing for STIs** |  |  |  |  |  |  |  |
| Fear of knowing status | 0.333 | 0.472 |  | 0.334 | 0.472 |  | 0.982 |
| Don't feel at risk | 0.110 | 0.314 |  | 0.096 | 0.295 |  | 0.472 |
| Not important to me | 0.201 | 0.401 |  | 0.197 | 0.398 |  | 0.813 |
| Cost | 0.620 | 0.486 |  | 0.606 | 0.489 |  | 0.489 |
| Didn't know where to go | 0.102 | 0.303 |  | 0.114 | 0.318 |  | 0.372 |
| Concerned about confidentiality | 0.016 | 0.126 |  | 0.015 | 0.120 |  | 0.786 |
| Negative attitude of healthcare worker | 0.005 | 0.073 |  | 0.003 | 0.052 |  | 0.335 |
| **Perceived risk for HIV** |  |  |  |  |  |  |  |
| High risk | 0.611 | 0.488 |  | 0.580 | 0.494 |  | 0.148 |
| Medium risk | 0.165 | 0.371 |  | 0.176 | 0.381 |  | 0.470 |
| Low risk | 0.080 | 0.271 |  | 0.090 | 0.286 |  | 0.385 |
| Not at risk | 0.118 | 0.323 |  | 0.140 | 0.347 |  | 0.134 |
| **Ever been tested for HIV** | 0.919 | 0.274 |  | 0.898 | 0.303 |  | 0.096 |
| SD: standard deviation |  |  |  |  |  |  |  |

**Appendix Figure 1: Weekly proportion reporting for testing (among the 10 randomly selected each week).**

**
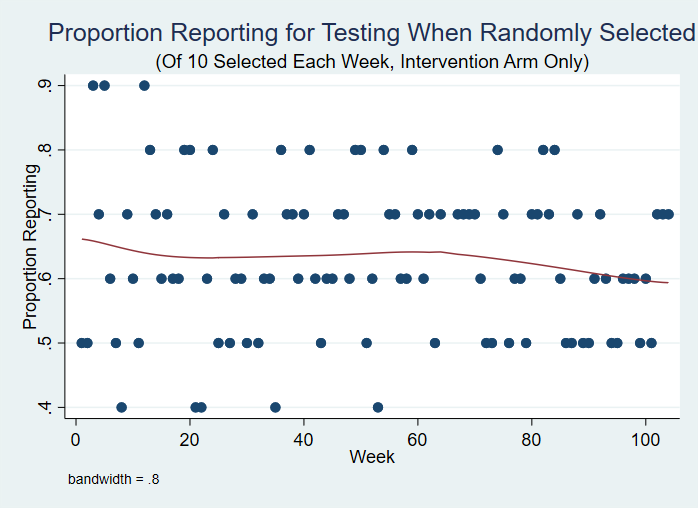
**

**Appendix Figure 2: Proportion testing STI positive each week.**

**
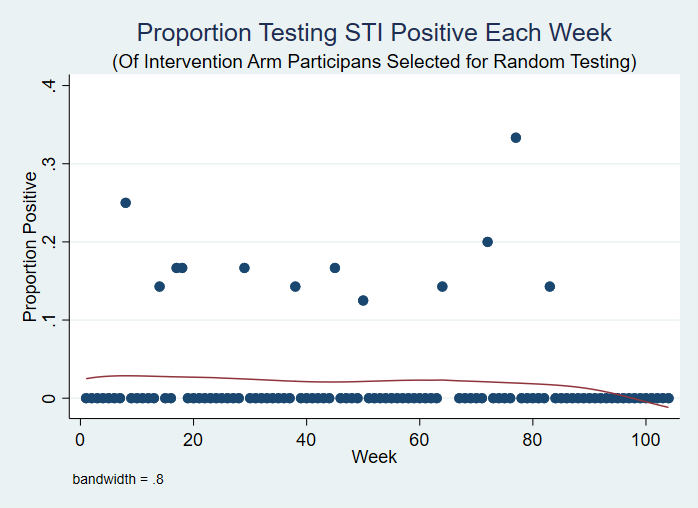
**

| **Supplemental Table 3. Baseline characteristics by endline response timing** |
| --- |
|  |

|  | **Early responders (n = 560)** | | **Late responders**  **(n = 557)** | | **p-value** (early vs. late) |
| --- | --- | --- | --- | --- | --- |
|  | Mean | SD | Mean | SD |  |
| **Respondent age** | 28.398 | 7.113 | 26.752 | 7.006 | 0.000 |
| **Education** |  |  |  |  |  |
| No formal education | 0.034 | 0.181 | 0.047 | 0.211 | 0.279 |
| Some primary complete | 0.084 | 0.278 | 0.054 | 0.226 | 0.047 |
| Primary complete | 0.582 | 0.494 | 0.603 | 0.490 | 0.474 |
| Some secondary complete | 0.136 | 0.343 | 0.117 | 0.321 | 0.339 |
| Secondary complete | 0.159 | 0.366 | 0.167 | 0.373 | 0.716 |
| High school | 0.002 | 0.042 | 0.002 | 0.042 | 0.997 |
| Vocational | 0.004 | 0.060 | 0.002 | 0.042 | 0.567 |
| University | 0.000 | 0.000 | 0.009 | 0.094 | 0.025 |
| **Lived in Dar es Salaam all their life** | 0.762 | 0.426 | 0.738 | 0.440 | 0.368 |
| Marital status |  |  |  |  |  |
| Never married | 0.704 | 0.457 | 0.738 | 0.440 | 0.202 |
| Divorced/separated | 0.259 | 0.438 | 0.226 | 0.419 | 0.203 |
| Currently married | 0.011 | 0.103 | 0.016 | 0.126 | 0.430 |
| Widowed | 0.014 | 0.119 | 0.011 | 0.103 | 0.598 |
| Cohabiting | 0.013 | 0.111 | 0.007 | 0.085 | 0.369 |
| **Has at least one child** | 0.780 | 0.414 | 0.707 | 0.455 | 0.005 |
| **Neighborhood poverty ranking (1-5)** | 2.691 | 0.663 | 2.673 | 0.706 | 0.653 |
| **Self-ranked social status (1-7)** | 3.807 | 1.571 | 3.633 | 1.475 | 0.057 |
| I**ncome** |  |  |  |  |  |
| Total income (30 days) Tsh - mean | 253742 | 196423 | 258666 | 382006 | 0.790 |
| Income from sex work (30 days) Tsh - mean | 236996 | 191110 | 239860 | 348298 | 0.866 |
| **Place of work** |  |  |  |  |  |
| Pub/bar | 0.431 | 0.496 | 0.378 | 0.485 | 0.076 |
| Guesthouse | 0.263 | 0.441 | 0.256 | 0.437 | 0.784 |
| Street | 0.076 | 0.265 | 0.081 | 0.273 | 0.738 |
| Night club/disco | 0.068 | 0.253 | 0.106 | 0.309 | 0.026 |
| Brothel | 0.070 | 0.256 | 0.068 | 0.253 | 0.906 |
| **Clients and condom use** |  |  |  |  |  |
| Number of clients /week | 8.360 | 6.750 | 8.383 | 6.975 | 0.956 |
| Amount earned with a condom | 14184 | 16688 | 13702 | 13338 | 0.604 |
| Amount earned without a condom | 20580 | 51555 | 18861 | 26741 | 0.540 |
| **Ever had an STI** | 0.063 | 0.243 | 0.068 | 0.252 | 0.711 |
| **Last time tested for STIs** |  |  |  |  |  |
| Within the last month | 0.023 | 0.151 | 0.023 | 0.151 | 0.989 |
| 1-2 months ago | 0.089 | 0.285 | 0.088 | 0.284 | 0.938 |
| 3-6 months ago | 0.157 | 0.364 | 0.171 | 0.376 | 0.545 |
| 6 months - 1 year ago | 0.129 | 0.335 | 0.120 | 0.326 | 0.675 |
| Over a year ago | 0.207 | 0.406 | 0.181 | 0.386 | 0.276 |
| Never | 0.395 | 0.489 | 0.417 | 0.493 | 0.457 |
| **Reasons for not testing for STIs** |  |  |  |  |  |
| Fear of knowing status | 0.330 | 0.471 | 0.336 | 0.473 | 0.895 |
| Don't feel at risk | 0.140 | 0.348 | 0.138 | 0.346 | 0.943 |
| Not important to me | 0.118 | 0.323 | 0.103 | 0.305 | 0.631 |
| Cost | 0.090 | 0.288 | 0.056 | 0.230 | 0.159 |
| Didn't know where to go | 0.068 | 0.252 | 0.056 | 0.230 | 0.602 |
| Concerned about confidentiality | 0.041 | 0.198 | 0.052 | 0.222 | 0.579 |
| Negative attitude of healthcare worker | 0.027 | 0.163 | 0.043 | 0.204 | 0.359 |
| **Perceived risk for HIV** |  |  |  |  |  |
| High risk | 0.630 | 0.483 | 0.591 | 0.492 | 0.174 |
| Medium risk | 0.145 | 0.352 | 0.185 | 0.389 | 0.070 |
| Low risk | 0.086 | 0.280 | 0.074 | 0.261 | 0.455 |
| Not at risk | 0.118 | 0.323 | 0.118 | 0.323 | 0.974 |
| **Ever been tested for HIV** | 0.925 | 0.264 | 0.912 | 0.284 | 0.429 |
| SD: standard deviation |  |  |  |  |  |

**Supplemental Table 4. Baseline characteristics, by treatment (Endline sample)**

|  | **Treatment (N = 609)** | | **Control (N = 508)** | | **p-value (treatment vs. control)** |
| --- | --- | --- | --- | --- | --- |
|  | Mean | SD | Mean | SD |  |
| **Respondent age** | 27.580 | 7.286 | 27.575 | 6.888 | 0.614 |
| **Education** |  |  |  |  |  |
| No formal education | 0.036 | 0.187 | 0.045 | 0.208 | 0.691 |
| Some primary complete | 0.077 | 0.267 | 0.059 | 0.236 | 0.545 |
| Primary complete | 0.576 | 0.495 | 0.612 | 0.488 | 0.596 |
| Some secondary complete | 0.133 | 0.340 | 0.118 | 0.323 | 0.533 |
| Secondary complete | 0.166 | 0.372 | 0.159 | 0.366 | 0.752 |
| High school | 0.002 | 0.041 | 0.002 | 0.044 | 0.665 |
| Vocational | 0.005 | 0.070 | 0.000 | 0.000 | 0.015 |
| University | 0.005 | 0.070 | 0.004 | 0.063 | 0.724 |
| **Lived in Dar es Salaam all their life** | 0.753 | 0.431 | 0.746 | 0.436 | 0.864 |
| Marital status |  |  |  |  |  |
| Never married | 0.727 | 0.446 | 0.713 | 0.453 | 0.388 |
| Divorced/separated | 0.236 | 0.425 | 0.250 | 0.433 | 0.532 |
| Currently married | 0.015 | 0.121 | 0.012 | 0.108 | 0.695 |
| Widowed | 0.010 | 0.099 | 0.016 | 0.125 | 0.061 |
| Cohabiting | 0.010 | 0.099 | 0.010 | 0.099 | 0.139 |
| **Has at least one child** | 0.726 | 0.446 | 0.766 | 0.424 | 0.996 |
| **Neighborhood poverty ranking (1-5)** | 2.688 | 0.687 | 2.675 | 0.681 | 0.658 |
| **Self-ranked social status (1-7)** | 3.730 | 1.524 | 3.709 | 1.529 | 0.571 |
| **Income** |  |  |  |  |  |
| Total income (30 days) Tsh - mean | 262542 | 337481 | 248676 | 257430 | 0.661 |
| Income from sex work (30 days) Tsh - mean | 245387 | 337041 | 230273 | 194052 | 0.153 |
| **Place of work** |  |  |  |  |  |
| Pub/bar | 0.406 | 0.491 | 0.403 | 0.491 | 0.945 |
| Guesthouse | 0.259 | 0.438 | 0.260 | 0.439 | 0.322 |
| Street | 0.081 | 0.273 | 0.075 | 0.264 | 0.253 |
| Night club/disco | 0.091 | 0.288 | 0.083 | 0.277 | 0.920 |
| Brothel | 0.068 | 0.251 | 0.071 | 0.258 | 0.871 |
| Clients and condom use |  |  |  |  |  |
| Number of clients /week | 8.363 | 6.784 | 8.382 | 6.959 | 0.606 |
| Amount earned with a condom | 13280 | 14287 | 13375 | 17015 | 0.890 |
| Amount earned without a condom | 18133 | 31327 | 18270 | 39207 | 0.936 |
| **Ever had an STI** | 0.069 | 0.254 | 0.061 | 0.240 | 0.219 |
| **Last time tested for STIs** |  |  |  |  |  |
| Within the last month | 0.018 | 0.133 | 0.030 | 0.169 | (.417 |
| 1-2 months ago | 0.090 | 0.287 | 0.087 | 0.282 | 0.250 |
| 3-6 months ago | 0.163 | 0.369 | 0.165 | 0.372 | 0.675 |
| 6 months - 1 year ago | 0.117 | 0.321 | 0.134 | 0.341 | 0.523 |
| Over a year ago | 0.205 | 0.404 | 0.181 | 0.385 | 0.493 |
| Never | 0.407 | 0.492 | 0.404 | 0.491 | 0.799 |
| **Reasons for not testing for STIs** |  |  |  |  |  |
| Fear of knowing status | 0.302 | 0.460 | 0.371 | 0.484 | 0.298 |
| Don't feel at risk | 0.129 | 0.336 | 0.151 | 0.359 | 0.180 |
| Not important to me | 0.109 | 0.312 | 0.112 | 0.316 | 0.085 |
| Cost | 0.093 | 0.291 | 0.049 | 0.216 | 0.424 |
| Didn't know where to go | 0.069 | 0.253 | 0.054 | 0.226 | 0.677 |
| Concerned about confidentiality | 0.036 | 0.187 | 0.059 | 0.235 | 0.139 |
| Negative attitude of healthcare worker | 0.040 | 0.197 | 0.029 | 0.169 | 0.518 |
| **Perceived risk for HIV** |  |  |  |  |  |
| High risk | 0.614 | 0.487 | 0.606 | 0.489 | 0.593 |
| Medium risk | 0.158 | 0.365 | 0.173 | 0.379 | 0.718 |
| Low risk | 0.090 | 0.287 | 0.067 | 0.250 | 0.006 |
| Not at risk | 0.113 | 0.317 | 0.124 | 0.330 | 0.380 |
| **Ever been tested for HIV** | 0.924 | 0.264 | 0.911 | 0.284 | 0.062 |
| SD: standard deviation |  |  |  |  |  |
